# Mixed attention ensemble for esophageal motility disorders classification

**DOI:** 10.1371/journal.pone.0317912

**Published:** 2025-02-14

**Authors:** Xiaofang Wu, Cunhan Guo, Junwu Lin, Zhenheng Lin, Qun Chen

**Affiliations:** 1 College of Electromechanical and Information Engineering, Putian University, Putian, Fujian, China; 2 School of Emergency Management Science and Engineering, University of Chinese Academy of Sciences, Beijing, Beijing, China; 3 New Engineering Industry College, Putian University, Putian, Fujian, China; 4 Putian Electronic Information Industry Technology Research Institute, Putian University, Putian, Fujian, China; Abu Dhabi University, UNITED ARAB EMIRATES

## Abstract

Esophageal motility disorders result from dysfunction of the lower esophageal sphincter and abnormalities in esophageal peristalsis, often presenting symptoms such as dysphagia, chest pain, or heartburn. High-resolution esophageal manometry currently serves as the primary diagnostic method for these disorders, but it has some shortcomings including technical complexity, high demands on diagnosticians, and time-consuming diagnostic process. Therefore, based on ensemble learning with a mixed voting mechanism and multi-dimensional attention enhancement mechanism, a classification model for esophageal motility disorders is proposed and named mixed attention ensemble(MAE) in this paper, which integrates four distinct base models, utilizing a multi-dimensional attention mechanism to extract important features and being weighted with a mixed voting mechanism. We conducted extensive experiments through exploring three different voting strategies and validating our approach on our proprietary dataset. The MAE model outperforms traditional voting ensembles on multiple metrics, achieving an accuracy of 98.48% while preserving a low parameter. The experimental results demonstrate the effectiveness of our method, providing valuable reference to pre-diagnosis for physicians.

## Introduction

**Fig 1:**
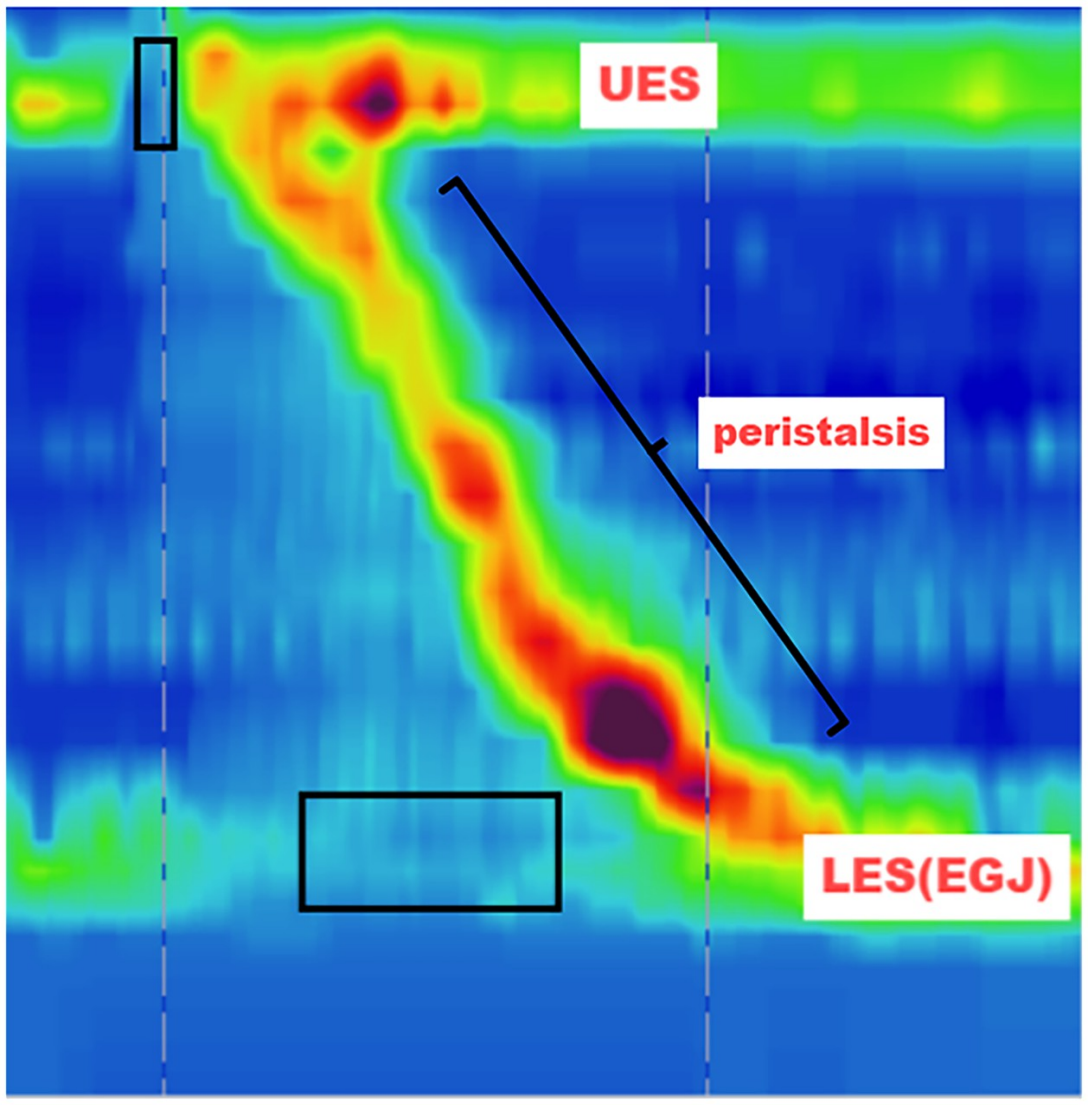
High-resolution esophageal manometry map of a normal swallowing process. Each point in the diagram corresponds to the pressure value at a specific location and time. The variation in pressure is depicted using color, with red representing high pressure, blue indicating low or no pressure, and green denoting moderate pressure. the upper and lower horizontal bands indicate the upper esophageal sphincter (UES) and the lower esophageal sphincter (LES), or the esophagogastric junction (EGJ). The upper and lower black boxes in the figure represent UES relaxation and LES relaxation, respectively. The slanted bands, depicted between the UES and LES, represent peristaltic activity.

The detection of esophageal motility disorders (EMDS) plays a crucial role in preventing esophageal diseases and improving patient survival rates. EMDS is caused by dysfunction of the upper/lower esophageal sphincter and/or defects in esophageal peristalsis, resulting in symptoms such as dysphagia and non-cardiac chest pain [1]. These disorders can be categorized into primary and secondary types. Given the systemic nature of secondary disorders affecting various organs, we primarily focus on primary diseases [2]. Primary indicates diseases originating with the esophagus itself [3]. According to the Chicago classification, primary diseases include esophagogastric junction outflow obstruction (EGJOO), diffuse esophageal spasm (DES), achalasia, hypercontractile esophagus (Jackhammer esophagus), and ineffective esophageal motility (IEM). High-resolution esophageal manometry (HREM) represents the gold standard for diagnosing EMDS [4]. HREM employs either water-perfused or solid-state catheters to uniformly measure pressures along the esophagus, generating dynamic color pressure topography as depicted in Fig 1. Utilizing the Chicago classification, HREM integrates these pressure metrics to provide a comprehensive esophageal motility diagnosis [5, 6].

Currently, the detection of EMDS primarily relies on manual diagnosis. Physicians assess the presence of esophageal motility abnormalities based on HREM swallow images and patient symptoms. This method requires extensive experience from physicians, leading to higher overall costs. To solve this issue, some research methods based on machine learning and deep learning have emerged. Teodora Surdea-Blaga [7] used the InceptionV3 deep learning model to accurately classify Integrated Relaxation Pressure (IRP) and employed the DenseNet201 CNN architecture to categorize images into five types of swallowing disorders. By combining results from these two models as input for a Chicago Classification decision tree, they achieved a top-1 accuracy and an F1 score of 86%. Wenjun Kou [8] used a long short-term memory (LSTM) deep learning model for training and evaluation, achieving an 88% accuracy in peristalsis classification. In the same year, Wenjun Kou [9] developed an automated diagnostic platform based on machine learning and artificial intelligence methods, which combination strategy employed weighted by precision scores to enhance accuracy. The above methods achieve high accuracy, but there are shortcomings in feature extraction, especially in image details, which affect the comprehensive performance of the model. To better extract image features and focus on crucial details, we propose a EMDS classification model based on a mixed voting mechanism and multi-dimensional attention enhancement, providing valuable reference to pre-diagnosis for physicians. The mixed voting mechanism primarily determines the final integration weight by synthesizing the performance of each model, which can more effective use the advantages of each model and improve the overall generalization ability. Additionally, the multi-dimensional attention mechanism, by focusing on different dimensions such as spatial and channel information, captures key details in images more comprehensively. This improves the accuracy and richness of feature representation, ultimately improving the robustness of the model.

Our contributions can be summarized as follows:

(1)By manually extracting and categorizing the esophageal swallow cloud images obtained from HREM, an esophageal motility dataset was constructed to facilitate deep learning analysis.
(2)A mixed voting mechanism was developed by combine four base models with multi-dimensional attention enhancement mechanism. The model weights are determined through the mixed voting mechanism, which were used for weighted integration to construct the MAE model.
(3) The final model achieved a recognition accuracy of 98.48% and has a model size of 74.6 MB, demonstrating excellent performance and suitability for classifying EMDS.

## Related work

### Esophageal motility disorders

Diagnosis of esophageal motility disorders (EMDS) involves several diagnostic techniques including endoscopic biopsy [10], barium-meal x-ray examination [11], HREM, acid reflux monitoring [12], and impedance planimetry [13]. HREM remains the primary diagnostic tool and standard for these disorders. Since diagnosis relies on the analysis of high-resolution manometry data, variability among assessors and potential interpretative errors can occur. Although automation and deep learning algorithms can help mitigate these errors, current research is limited, and such technologies are not yet applied in clinical practice. In 2017, Alessandro Frigo [14] developed an automated diagnostic system for EMDS that reduces human intervention in data processing, achieving correct diagnoses for up to 86% of cases. Subsequently, a series of studies expanded on EMDS. Alissa Jell [15] employed supervised machine learning algorithms to develop and evaluate an automated swallow recognition and classification system, which significantly reduced the core assessment time for a 24-hour long-term HREM, shortening it from 3 days to just 11 minutes. Wenjun Kou [16] utilized over 32,000 raw swallowing data samples to develop a generative model using a variational autoencoder (VAE) method. In 2022, Stefan Lucian Popa [17] used an Inception V3 CNN model, pre-trained on ImageNet, to categorize EMDS into ten categories, achieving an overall accuracy exceeding 93%. Two years later, Stefan Lucian Popa [18] utilizing the Gemini tool, developed a deep learning model for diagnosing EMDS from high-resolution manometric images, achieving an overall accuracy of 89%. In the same year, Geiger [19] introduced a deep learning-based method for detecting swallowing events, which enabled the accurate identification of swallowing and secondary non-deglutition-induced esophageal motility disorders in long-term (up to 24 hours) high-resolution manometry (LTHRM) data, reaching an accuracy of over 94%.

To obtain important channel spatial information, we propose the MAE model, which integrates ensemble learning with a mixed voting mechanism and multi-dimensional attention enhancement mechanism. This model used preprocessed swallowing images as inputs for training and testing and demonstrated a high performance with a recognition accuracy of 98.48%.

### Multi-dimensional attention enhancement mechanism

In medical image processing tasks, due to the complexity of image details, and to allow networks to learn image features more finely and focus on important feature information, more and more models are beginning to use multi-dimensional attention enhancement mechanisms to improve recognition accuracy. RuoXi Qin [20] combined a 3D DenseNet network with multi-dimensional (channel and spatial) attention mechanisms to further enhance the extraction of fine-grained features, ultimately achieving an area under the ROC curve of 0.92 (balanced accuracy = 0.72) and a more focused network attention point, enabling noninvasive diagnosis of lung cancer. Gongping Chen [21] introduced a hybrid adaptive attention module (HAAM) and developed an adaptive attention U-Net (AAU-Net) for automatically stable segmentation of breast lesions from ultrasound images. This model can adaptively select more robust representations in both channel and spatial dimensions, demonstrating good generalization capability. Luyang Cao [22] proposed MDAG-Net (Multi-dimensional Attention Gate Network), which integrates spatial, channel, and multi-dimensional feature map attention mechanisms to capture global semantic information across spatial and channel dimensions, filter redundant information in shallow feature maps, and achieve better segmentation of small target regions compared to the U-Net network. Guohao Xu [23] employed the LE-NeXt framework, which incorporates multi-dimensional spatial attention and multi-scale feature extraction, integrating lightweight convolution and attention mechanisms into an encoder-decoder model. This approach enhances stage-specific feature extraction while ensuring efficiency, achieving an IoU accuracy of 89.8%. Shidong Zhang [24] proposed a multi-scale attention residual network (MAResNet) for diagnosing pulmonary tuberculosis (PTB) in patients using computed tomography (CT) images. By integrating a residual module with the convolutional block attention module (CBAM), the network enhances its focus on key features, improves the image feature resolution, expands the network’s global receptive field, and effectively captures the local details of pulmonary nodules. The results demonstrated an overall accuracy of 94% in PTB classification, offering significant potential for aiding doctors in making more accurate diagnoses. Dequn Zhao [25] developed the MDA-Unet model, built upon the U-Net architecture, for retinal image segmentation. By incorporating mixed pooling and a multi-scale attention mechanism, the model achieved promising results in the segmentation of fundus blood vessels.

Research on the application of multi-dimensional attention enhancement mechanisms for EMDS classification is currently limited. To enhance feature extraction from swallowing images and improve the accuracy of EMDS recognition, we have incorporated a multi-dimensional attention enhancement module into four pre-trained base models. This integration aims to focus more on critical channel and spatial information.

### Ensemble learning

As deep learning increasingly dominates the field of artificial intelligence, ensemble learning has demonstrated exceptional performance in enhancing system generalization capabilities [26]. Ensemble learning is a machine learning method which select a set of individual learners and combining them using specific strategies to improve performance in classification and prediction tasks. Individual learner is typically trained using existing AI algorithms. When the same type of learner is used, it is referred to as homogeneous ensemble learning; when different types are used, it is known as heterogeneous ensemble learning [27].

We explore a heterogeneous ensemble learning approach by integrating four base models. Compared to individual learners, ensemble learning generally offers higher accuracy, and its application in medicine is expanding, but studies on EMDS remain limited. Azadeh Bayani [28] employed ensemble learning methods, such as Catboost and XGB classifiers, to predict the esophageal variceal (EV) grade in a dataset of 490 cirrhotic patients, selecting the most effective predictors for EV grade. The Catboost model achieved 100% accuracy for all prediction targets, while the XGB classifier reached an overall accuracy of 91.02%. Qiaosen Su [29] applied transfer learning with EfficientNetB3, DenseNet, and Xception, using the modified and fine-tuned backbone networks as feature extractors in ensemble learning, achieving excellent performance in detecting gastrointestinal diseases. Esra Sivari [30] proposed a set of hybrid stacking ensemble models for the detection and classification of gastrointestinal systems. In the first level of the stacking ensemble method, 5-fold cross-validation was applied to three new CNN models to obtain predictions; in the second level, selected machine learning classifiers were trained based on these predictions, achieving an accuracy of 98.42% on the KvasirV2 dataset. Hemalatha Gunasekaran [31] developed an endoscopic image classification model for the gastrointestinal tract, using DenseNet201, InceptionV3, and ResNet50 as individual classifiers. By combining the predictions of these classifiers using both model averaging and weighted averaging methods, the weighted averaging ensemble outperformed both the model averaging ensemble and individual models, achieving an accuracy of 95.00%. Faysal Ahamed [32] proposed a novel model for accurately classifying gastrointestinal (GI) diseases, achieving an accuracy of 88.12 ± 0.332%. This model integrates a parallel Depthwise Separable CNN (PD-CNN) feature extractor and a Pearson Correlation Coefficient (PCC) feature selector with an Ensemble ELM (EELM) classifier, demonstrating excellent classification performance.

## Dataset collection and processing

**Fig 2:**
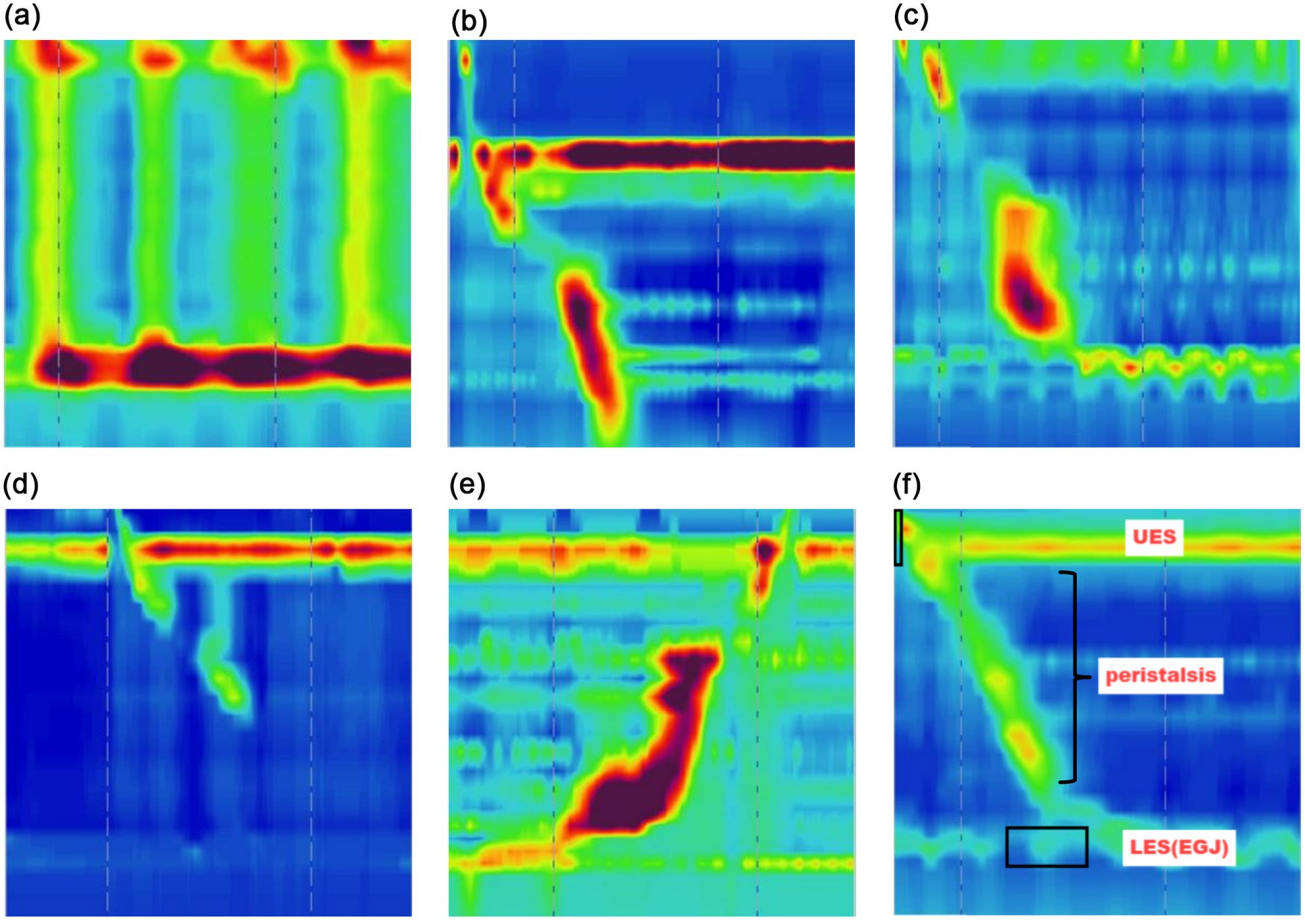
Classification of EMDS. (a) represents the Achalasia type II category, where increased LES pressure and continuous high pressure in the esophageal body are observed, with a complete absence of normal esophageal peristalsis. (b) shows the DES category, characterized by inconsistent contraction waves within the esophageal body, along with high-amplitude and intermittent pressure waves. (c) represents the EGJOO category. Here, the LES pressure is markedly elevated compared to the normal relaxed state. Although the peristaltic waves in the esophageal body may appear normal, there is a significant increase in pressure when passing through the esophagogastric junction. High-pressure regions primarily occur at the junction between the lower esophagus and the stomach, indicating obstruction and impaired emptying. (d) depicts the IEM category, where the lack of normal esophageal peristalsis leads to impaired food transit. (e) shows the Jackhammer category, characterized by excessively strong and frequent contraction waves within the esophageal body. These contractions often manifest as localized high-pressure areas, with pressure values significantly higher than those seen in normal esophageal contractions. These waves also exhibit intermittent and strongly repetitive patterns. (f) represents normal esophageal swallowing.

The image data used in this study were obtained from HREM recordings. The original data consisted of continuous sequences, each containing 20 frames that captured the swallowing conditions of the esophagus at different time points. To process these data, we manually extracted individual frames, converting the continuous sequences into separate images. This resulted in a dataset of 2,315 swallowing samples, which were resized to 224 × 224 pixels for consistency. The samples were then categorized into six groups based on the corresponding clinical conditions: Achalasia, DES, EGJOO, IEM, Jackhammer, and normal. Due to the limited size of the dataset, the Achalasia category primarily focuses on type II. The representative swallowing images for each category are illustrated in Fig 2. The data were then split into training, validation, and test sets at a ratio of 6:2:2. To enhance model generalization and reduce overfitting, data augmentation was applied to the training images.

Data augmentation expanded the original training images to eight times their initial quantity. The augmentation methods included: random brightness enhancement ranging from 1.6 to 2.0 times; random contrast and saturation enhancement from 2.1 to 2.5 times; random rotation within -10 to 10 degrees; random scaling between 0.5 and 0.9; and the addition of Gaussian and salt-and-pepper noise. The distribution of the dataset is detailed in Table 1.

**Table 1:** Statistical summary of image counts for each dataset.

Category	Original Dataset			Augmented Dataset		
	Train	Val	Test	Train	Val	Test
Achalasia II	108	35	35	864	35	35
DES	205	67	67	1640	67	67
EGJOO	144	48	48	1152	48	48
IEM	291	96	96	2328	96	96
Jackhammer	60	20	20	480	20	20
Normal	585	195	195	4680	195	195

## Method

### Overview of MAE

To obtain richer features and improve generalization capability of the model, we propose a model named MAE, which integrates ensemble learning by a mixed voting mechanism and multi-dimensional attention enhancement mechanism. The current mainstream models pre-trained on ImageNet were trained and tested respectively. By considering both the model size and accuracy, we identified the four highest-performing models: ResNet18, MobileNetV3_Small, GoogLeNet, and EfficientNetB0, which were chosen as the base models for our study. Since the swallowing images obtained from HREM contain dynamic information about the swallowing process and are sensitive to both spatial and channel details, we incorporated a channel and spatial dimensional attention enhancement(CSAE) module into the final decision layers of four base models. This integration aims to capture critical spatial and channel information effectively.

**Fig 3:**
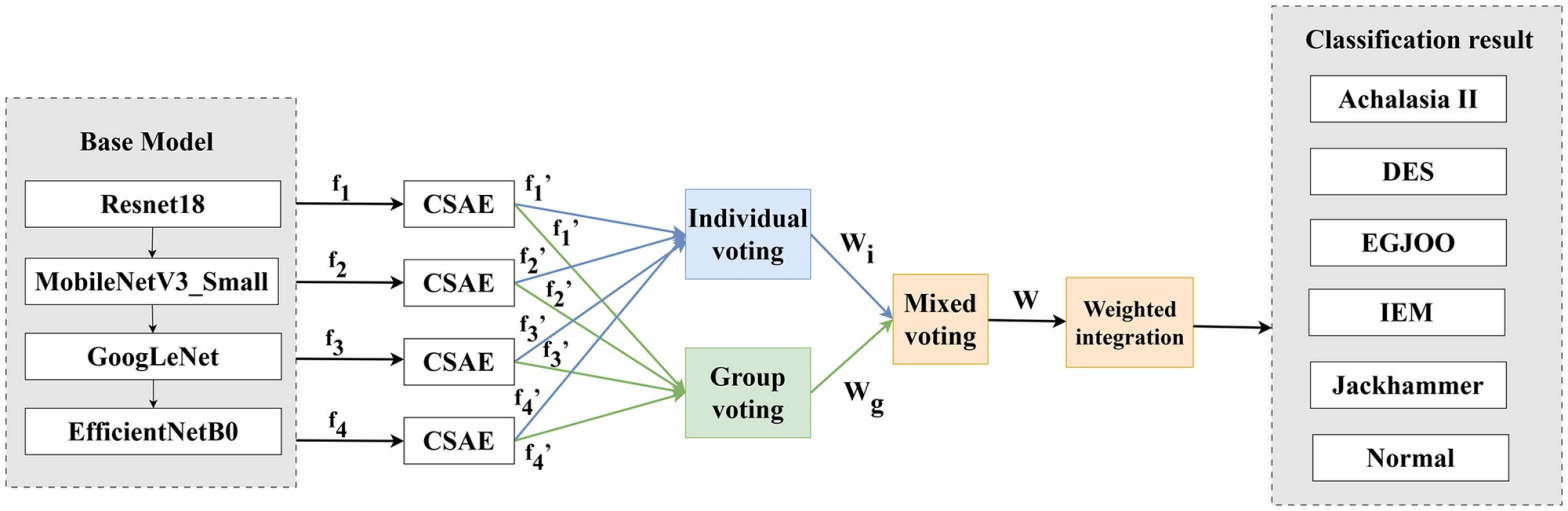
The main framework of the MAE model. The figure illustrates how each basic model extracts features, and how these features are subsequently weighted through a mixed voting integration method to produce the final classification result.

Ensemble learning commonly uses voting mechanisms for classification, but traditional methods often base weights solely on model accuracy, which can be limited. To solve this problem, we propose a mixed voting mechanism: each of the four basic models is used separately as a primary model and integrated with the other three models through weights. The weights are updated during the training process to determinate four sets of weights. These four sets of weights are then integrated to produce the final weights, which represent the individual voting weights. During the training process, all base models continuously update their voting weights, producing group voting weights. The mixed voting mechanism combines individual and group voting weights to produce the final model weights. The base models are multiplied by their respective weights derived from the mixed voting mechanism after multi-dimensional attention enhancement, then trained and tested to obtain classification results. The main framework of the model is illustrated in Fig 3.

Here, *f*_*i*_(where i = 1,2,3,4) denotes features extracted from the ResNet18, MobileNetV3_Small, GoogLeNet, and EfficientNetB0 models, respectively. *f*_*i*_’ represents the outputs of each model after the module of CSAE, as shown in Eq (1). *W*_*i*_ indicates the weights derived from individual voting, *W*_*g*_ represents the weights derived from group voting, and W denotes the final weights obtained through the mixed voting mechanism, as described in Eq (2), where *β*_1_ is the optimal coefficient for weighting group votes, derived from ablation studies.

$$\begin{array}{c} \begin{array}{cc} & f_{i}^{\prime }=\text{CSAE}(f_{i}) \end{array} \end{array}$$ (1)

$$\begin{array}{c} W=\beta_{1}\cdot W_{g}+(1-\beta_{1})\cdot W_{i} \end{array}$$ (2)

### Multi-dimensional attention enhancement module

**Fig 4:**
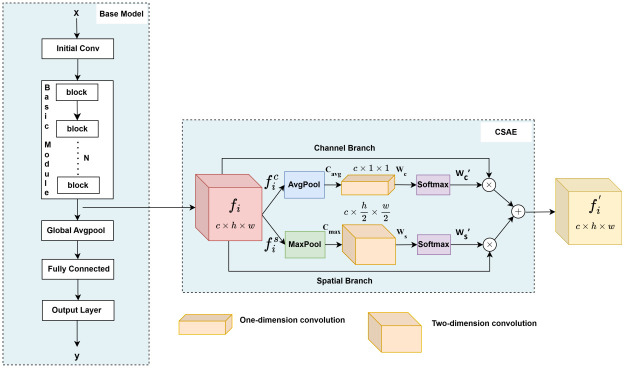
The CSAE module for each base model. This figure illustrates the process of attention enhancement applied by the CSAE module to each base model.

In the experiment, a CSAE module was added in front of the decision level of the four basic models to better capture spatial and channel features from image data. The multi-dimensional attention enhancement process of CSAE for each base model is shown in Fig 4.

Initially, the preprocessed 224 × 224 × 3 image data was through an initial convolution (Initial Conv) for preliminary feature extraction and downsampling. The resulting features are then passed to a basic module (Basic Module), each of which consists of several blocks, which can be the residual blocks [33], the inverted residual blocks [34], the inception blocks [35], or the MBConv blocks [36]. This yields a feature map *f*_*i*_, where i = 1,2,3,4 represents the feature maps obtained from the four base models after the basic module. Subsequently, feature map *f*_*i*_ is input to the CSAE module for both channel and spatial attention enhancement. Average and max pooling operations are applied to generate two channel vectors *C*_*avg*_ and *C*_*max*_ to capture key global and local information in the feature map, as shown in Eq (3), where $f_{i}^{c}$ represents features along the channel dimension and $f_{i}^{s}$ represents features along the spatial dimension.

$$\begin{array}{c} C_{\text{avg}\;},C_{\text{max}}=\text{AvgPool}(f_{i}^{c}),\text{MaxPool}(f_{i}^{s}) \end{array}$$ (3)

The obtained channel vectors are processed through 1D convolution operations to produce channel attention weights *W*_*c*_. Subsequently, the results through softmax normalization to yield the final channel attention weights *W*_*c*_’, as illustrated in Eqs (3) and (4). Here, Conv1(·) denote 1D convolution operations.

$$\begin{array}{c} W_{c}=\text{Conv1}(C_{\text{Avg}\;}) \end{array}$$ (4)

$$\begin{array}{c} W_{c}^{\prime }=\text{softmax}(W_{c}) \end{array}$$ (5)

Similarly, spatial attention weights *W*_*s*_’ are obtained using the same method applied in the spatial dimension, capturing critical spatial information. Then the obtained spatial attention weight and channel attention weight are multiplied by the input features $f_{i}^{c}$ and $f_{i}^{s}$ respectively, and added together to enhance the important channel and spatial features in the image. The final output feature is *f*_*i*_’, as shown in Eq (6).

$$\begin{array}{c} {\text{fi}}^{\prime }=(W_{c}^{\prime }\cdot f_{i}^{c})+(W_{s}^{\prime }\cdot f_{i}^{s}) \end{array}$$ (6)

### Voting mechanism

The proposed mixed voting mechanism combines individual and group voting mechanisms. The individual voting mechanism is integrated and trained by specifying one model as the primary model and the others as secondary models in each iteration. During training, weights are adjusted based on the accuracy of each model, resulting in four weight sets: *W*_1_, *W*_2_, *W*_3_, and *W*_4_. The calculation formula of individual vote weight *W*_*i*_ is shown in Eq (7), where *W*_*i*_ is the weighted sum of the four weight sets, and *α*_1_, *α*_2_, *α*_3_ and *α*_4_ are the corresponding weight coefficients, and the sum is always 1.

$$\begin{array}{c} W_{i}=\alpha_{1}\cdot W_{1}+\alpha_{2}\cdot W_{2}+\alpha_{3}\cdot W_{3}+\alpha_{4}\cdot W_{4} \end{array}$$ (7)

The formulas for calculating these coefficients are provided in Eq (8). Here, *A*_*i*_ represents the recognition accuracy of i th model and $\sum_{j=1}^{n}e^{A_{j}}$ is calculated as the exponent sum of the accuracies in all the base models, where n denotes the total number of base models.

$$\begin{array}{c} \alpha_{i}=\frac{e^{A_{i}}}{\sum_{j=1}^{n}e^{A_{j}}} \end{array}$$ (8)

The group voting weight is obtained through a weighted integration process, starting with assigning an initial weight *W*_*gi*_, where i = 1,2,3,4 to each base model randomly. During the training process, weights are updated to $W_{gi}^{*}$ based on testing accuracy, * represents the updated weights to distinguish the initial weights. The best set of weights is eventually saved as the group voting weight *W*_*g*_. The calculation formulas are shown in Eqs (9) and (10). Here, f($W_{g}^{*}$) represents the accuracy obtained using the weights $W_{g}^{*}$ on the validation set. During validation, the set of weights that yields the highest accuracy is selected as the group voting weight *W*_*g*_.

$$\begin{array}{c} W_{g_{i}}^{*}=W_{g_{i}}\times \frac{A^{i}}{\sum_{j=1}^{n}A^{j}} \end{array}$$ (9)

$$\begin{array}{c} W_{g}=\text{arg}\;\underset{W_{g}^{*}}{\text{max}}f(W_{g}^{*}) \end{array}$$ (10)

The mixed voting mechanism combines the group voting weight *W*_*g*_ and the individual voting weight *W*_*i*_ using artificially randomly assigned weight coefficients *β*_1_ and *β*_2_. Experiments determined through ablation studies that the best classification performance is achieved when *β*_1_: *β*_2_ = 4:1, for more detail, you can refer to section 5.3.1.

## Experiment

### Experiment setting

The model in this experiment was trained using the PyTorch deep learning framework. The hardware specifications for testing were as follows: the CPU was an Intel(R) Xeon(R) Platinum 8352V with a clock speed of 2.10 GHz and 60 GB of RAM. The GPU was an RTX 4090D with 24 GB of video memory. The operating system was Ubuntu 18.04, with PyTorch version 1.7.0 and Python version 3.8. Hyperparameters for the network were adjusted during the experiment. The total number of training epochs was set to 100, with a batch size of 16 per epoch. A learning rate of 0.001 was used to ensure stable convergence. The Cross-entropy loss function and the adaptive moment estimation (Adam) were employed to accelerate convergence.

To objectively evaluate the performance of the MAE model on the esophageal motility dataset, we used five metrics: accuracy, error rate, precision, recall, and F1 score. The definitions of these metrics are as follows:

$$\begin{array}{c} Accuracy=\frac{\text{TP}+\text{TN}}{\text{TP}+\text{TN}+\text{FP}+\text{FN}} \end{array}$$ (11)

$$\begin{array}{c} Error\;Rate=1-Accuracy \end{array}$$ (12)

$$\begin{array}{c} Precision=\frac{\text{TP}}{\text{TP}+\text{FP}} \end{array}$$ (13)

$$\begin{array}{c} Recall=\frac{\text{TP}}{\text{TP}+\text{FN}} \end{array}$$ (14)

$$\begin{array}{c} F1=\frac{2\;\text{Precision}\;\times \;\text{Recall}\;}{\;\text{Precision}\;+\;\text{Recall}\;} \end{array}$$ (15)

True Positives (TP) refer to the number of instances where the actual class is correctly classified as that class by the model. False Negatives (FN) are the instances where the actual class is that category, but the model incorrectly classifies them as belonging to other categories. False Positives (FP) denote the cases where the actual class does not belong to that category, yet the model mistakenly classifies them as that category. True Negatives (TN) are the instances where the actual class does not belong to that category, and the model correctly classifies them as not belonging to that category.

### Comparison with other methods

To better evaluate the performance of the model, we compared the MAE model with the base models and other current mainstream classification models. The results are presented in Table 2. The MAE model demonstrates excellent performance, achieving an accuracy of 98.48%. Compared to the four base models, the MAE model shows significant improvements across various metrics. Specifically, compared to the best-performing base model, ResNet18, the MAE model reduces the error rate by 0.44%, increases precision by 0.48%, improves recall by 1.22%, and enhances the F1 score by 0.85%. Among other popular models, although the Swin Transformer model performed better than the base models, the MAE model outperforms it across all metrics and is smaller in size (74.6M), which is 257M less than the Swin Transformer model. In summary, the MAE model offers high accuracy and is well-suited for EMDS classification tasks.

**Table 2:** Comparison results of different models (models marked with * are base model).

Model	Accuracy(%) ↑	Error rate(%) ↓	Precision (%) ↑	Recall(%) ↑	F1 Value(%) ↑	Parameter size(M) ↓
ResNet18 [2015]*	98.04	1.96	98.05	98.04	98.04	42.70
MobileNetV3_Small [2019]*	96.96	3.04	96.95	96.96	96.93	5.95
GoogLeNet [2014]*	96.74	3.26	96.74	96.74	96.72	21.50
EfficientNetB0 [2019]*	97.18	2.82	97.17	97.18	97.16	15.60
DenseNet161 [2017] [37]	97.61	2.39	97.63	97.61	97.60	102.00
Vgg16 [2014] [38]	96.31	3.69	96.44	96.31	96.30	512.30
**MAE[Ours]**	**98.48**	**1.52**	**98.53**	**99.26**	**98.89**	**74.60**
Swin Transformer [2021] [39]	98.26	1.74	98.27	98.26	98.25	331.60
Vision Transformer [2020] [40]	96.96	3.04	96.99	96.96	96.55	327.40
Pyramid Vision Transformerv2 [2022] [41]	97.39	2.61	97.40	97.39	97.37	172.80
RepViT [2023] [42]	97.18	2.82	97.19	97.18	97.17	8.50

↑: Indicates that a higher value is better,

↓: Indicates that a lower value is better.

Table 3 presents the recognition results of the MAE model for various esophageal motility disorder categories. The results show that the MAE model demonstrates excellent performance in identifying EGJOO and Jackhammer disorders, achieving 100% across all metrics. Both Achalasia II and IEM also reach 100% precision, recall, and F1 score; however, Achalasia II shows a slightly higher recognition accuracy at 97.14% compared to IEM. For DES and normal categories, the model achieves a 100% accuracy rate. Nevertheless, DES exhibits higher precision, recall, and F1 score compared to the normal category. Overall, the MAE model exhibits high accuracy in recognizing all categories, showing good performance.

**Table 3:** Performance of the MAE model on different esophageal motility disorder categories.

Class	Accuracy(%) ↑	Precision (%) ↑	Recall (%) ↑	F1 Value(%) ↑
Achalasia II	97.14	100.00	100.00	100.00
DES	100.00	97.08	98.53	97.80
EGJOO	100.00	100.00	100.00	100.00
IEM	93.75	100.00	100.00	100.00
Jackhammer	100.00	100.00	100.00	100.00
Normal	100.00	94.12	97.01	95.55

↑: Indicates that a higher value is better,

↓: Indicates that a lower value is better.

**Fig 5:**
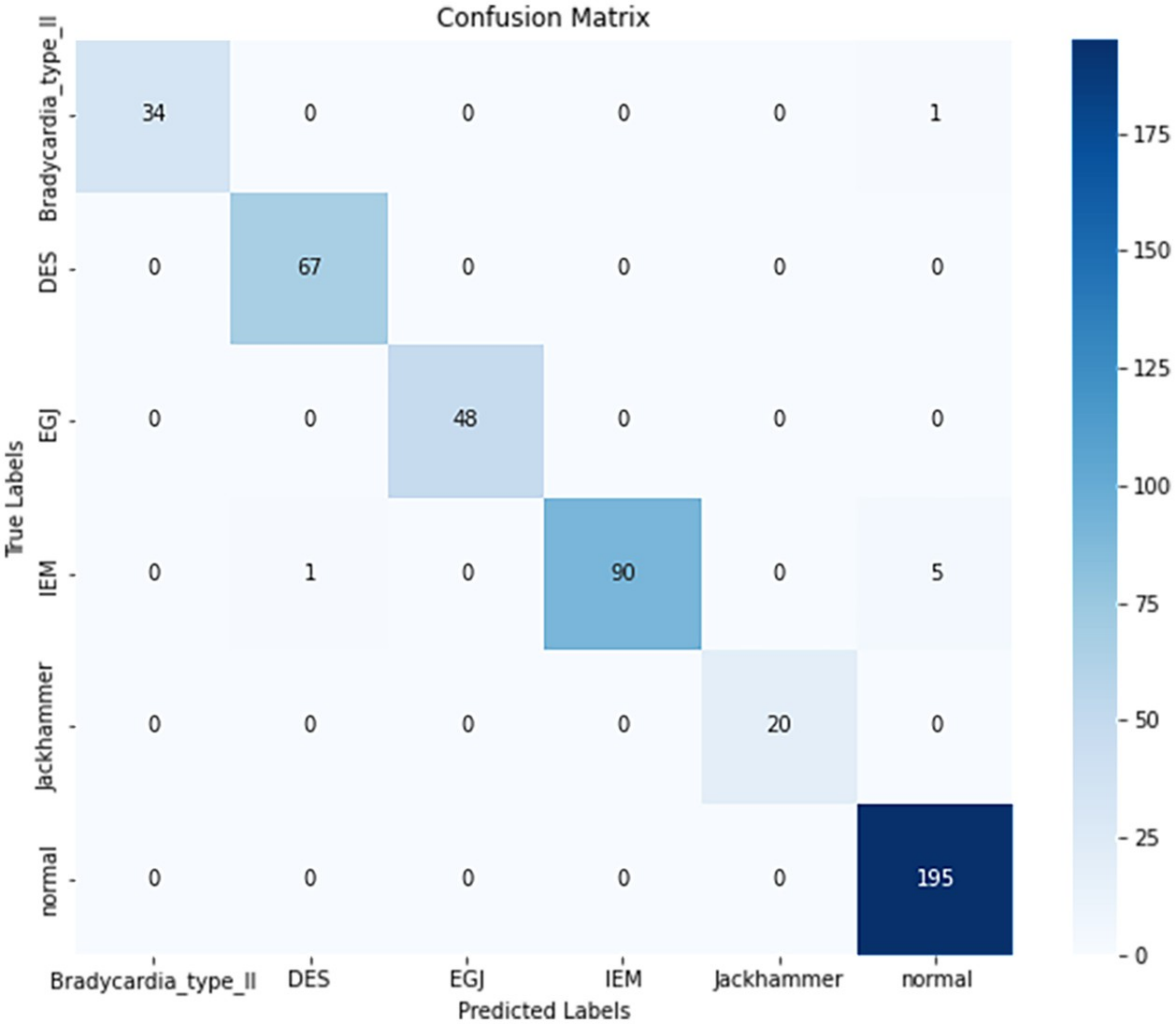
Confusion matrix for classification testing with the MAE model. The vertical axis represents the true categories, while the horizontal axis represents the predicted categories. The diagonal elements indicate the number of instances where the true and predicted categories match.

Fig 5 shows the confusion matrix obtained from classification testing with the MAE model. The confusion matrix indicates that the MAE model misclassified a total of 7 samples. Specifically, 1 sample of Achalasia was incorrectly classified as normal, 1 sample of IEM was misclassified as DES, and 5 samples of IEM were incorrectly classified as normal. These misclassifications may be attributed to the complexity of esophageal motility analysis, where some data exhibit similar movement patterns. Additionally, the limited size of the dataset may have prevented the complete learning of image features, leading to these classification errors.

### Parameter finetuning and ablation study

#### Parameter finetuning

To determine the optimal weighting coefficients for group voting and individual voting in the mixed voting mechanism, we compared the test results of various weighting coefficients. The first column of Table 4 shows the weight coefficient *β*_1_ for group voting weights, while the remaining four columns present the corresponding evaluation metrics. The weighting coefficient *β*_2_ for individual voting weights is defined in Eq (16). The results indicate that a *β*_1_ value of 0.9 yields the highest recognition accuracy at 98.70%. When *β*_1_ is 0.1 or 0.2, the recognition accuracy is 98.48%, which is slightly lower than that achieved with *β*_1_ of 0.9. However, *β*_1_ of 0.2 has a higher precision, recall and F1 score than *β*_1_ of 0.1. Despite the highest accuracy with *β*_1_ of 0.9, its precision, recall, and F1 score are notably lower than *β*_1_ of 0.2. Therefore, considering all metrics, *β*_1_ of 0.2 is chosen as the final weighting coefficient for group voting. In this setting, *β*_2_ is 0.8, which provides the best overall performance for the model. Specifically, when *β*_1_ is 0, representing individual voting ensemble, the recognition accuracy is 96.96%. Conversely, when *β*_1_ is 1, indicating the group voting ensemble, the accuracy increases to 97.83%. It is evident that our proposed mixed voting ensemble markedly surpasses both individual and group voting ensembles in all performance metrics.

$$\begin{array}{c} \beta_{2}=1-\beta_{1} \end{array}$$ (16)

**Table 4:** Comparison of different weight coefficients for group voting in the mixed voting mechanism.

Group voting weight coefficient(*β*_1_)	Accuracy(%)↑	Precision (%) ↑	Recall (%) ↑	F1 Value(%) ↑
0.0	96.96	96.61	98.27	97.42
0.1	98.48	98.02	99.00	98.50
**0.2**	**98.48**	**98.53**	**99.26**	**98.89**
0.3	97.83	97.04	98.50	97.76
0.4	97.61	96.36	98.13	97.23
0.5	98.05	97.04	98.50	97.76
0.6	97.61	96.81	98.37	97.57
0.7	98.05	97.32	98.64	97.97
0.8	97.40	96.86	98.39	97.60
0.9	98.70	98.38	99.18	98.78
1.0	97.83	97.87	97.83	97.80

↑: Indicates that a higher value is better,

↓: Indicates that a lower value is better.

#### Ablation study

To further validate the effectiveness of the MAE model, we conducted an ablation study by decoupling the CSAE module and the mixed voting mechanism while maintaining the same experimental setup as described in section 5.1. The results are presented in Table 5. In this table, “Average weighted voting” refers to assigning a weight of 0.25 to each of the four base models and integrating them. “Standard weighted voting” involves training each base model separately and determining the weighting ratio based on the testing accuracy of each model.

**Table 5:** Ablation analysis with MAE and voting mechanisms.

Model	Accuracy(%) ↑	Precision (%) ↑	Recall (%) ↑	F1 Value(%) ↑	Parameter size(M) ↓
average weighted voting	98.04	98.05	98.04	98.04	85.90
standard weighted voting	98.48	98.49	98.48	98.46	85.90
individual voting	97.40	97.16	98.55	97.84	74.60
individual voting + CSAE	96.96	96.61	98.27	97.42	74.60
group voting	97.39	97.45	97.39	97.37	74.60
group voting +CSAE	97.83	97.87	97.83	97.80	74.60
mixed voting	98.48	98.22	99.10	98.66	74.60
**mixed voting +CSAE(ours)**	**98.48**	**98.53**	**99.26**	**98.89**	**74.60**

↑: Indicates that a higher value is better,

↓: Indicates that a lower value is better.

From Table 5, it is evident that, without the CSAE multi-dimensional attention enhancement module, the mixed voting mechanism significantly enhances accuracy, precision, recall, and F1 score compared to both the individual and group voting mechanisms. When the CSAE module is included, the ensemble using individual voting shows a decline in all performance metrics, despite unchanged model parameters size. This decline may be attributed to the enhancement strategy of CSAE module, which might not be well-suited for the individual voting mechanism, potentially leading to excessive feature enhancement and thus adversely affecting the final classification results. In contrast, integrating CSAE with the group voting mechanism results in minor improvements across all metrics. With the CSAE module, the mixed voting mechanism maintains an accuracy of 98.48% but shows increases of 0.31% in precision, 0.16% in recall, and 0.23% in F1 score. Our proposed MAE model integrates the advantages of both group and individual voting mechanisms, incorporating the CSAE module to extract crucial channel and spatial features. This approach enhances the attention of model to key areas and feature channels within the images, allowing for more effective capture of critical information and improving classification accuracy. Compared to traditional average weighted voting, the MAE model outperforms in all metrics and has a smaller model size. Although standard weighted voting also achieves a high accuracy of 98.48%, it falls short of the MAE model in terms of precision, recall, and F1 score.

## Conclusion

In this study, we introduce a new method for classifying esophageal motility disorders, namely the MAE model. This model is trained and evaluated on self-built datasets. The MAE involves four pre-trained base models and leverages multi-dimensional attention enhancement strategies for feature encoding. A mixed voting mechanism is also employed for ensemble learning to achieve better feature fusion. The model achieves competitive results across multiple metrics, particularly an accuracy of 98.48%, with a compact size of 74.6 MB. This represents a reduction in model parameters by 11.3 MB compared to traditional voting ensemble methods. The experimental results demonstrate the effectiveness and efficiency of our method, and we believe our work will assist clinicians in the preliminary diagnosis of esophageal motility disorders and improve diagnostic efficiency.

## References

[1] Hoshikawa Yoshimasa and Iwakiri Katsuhiko, Esophageal Motility Disorders: Diagnosis and Treatment Strategies, *Digestion*, vol. 105, no. 1, pp. 11–17, 2024. S. Karger AG. doi: 10.1159/000533347 37634495

[2] Nehra Avinash K., Sheedy Shannon P., Daniel Johnson C., Flicek Kristina T., Venkatesh Sudhakar K., Heiken Jay P., et al., Imaging review of gastrointestinal motility disorders, *Radiographics*, vol. 42, no. 7, pp. 2014–2036, 2022. Radiological Society of North America. doi: 10.1148/rg.220052 36206184

[3] Mittal Ravinder and Vaezi Michael F., Esophageal motility disorders and gastroesophageal reflux disease, *New England Journal of Medicine*, vol. 383, no. 20, pp. 1961–1972, 2020. Mass Medical Soc. doi: 10.1056/NEJMra2000328 33176086

[4] Patel Dhyanesh A., Yadlapati Rena, and Vaezi Michael F., Esophageal motility disorders: current approach to diagnostics and therapeutics, *Gastroenterology*, vol. 162, no. 6, pp. 1617–1634, 2022. Elsevier. doi: 10.1053/j.gastro.2021.12.289 35227779 PMC9405585

[5] Meng DAI, Jie WANG, Xiaomei WEI, Chao LI, Zitong HE, Zulin DOU, To evaluate the motility of the esophageal phase of swallowing among brainstem stroke survivors, *Chinese Journal of Physical Medicine and Rehabilitation*, pp. 13–17, 2020.

[6] Yadlapati Rena, High-resolution esophageal manometry: interpretation in clinical practice, *Current opinion in gastroenterology*, vol. 33, no. 4, pp. 301–309, 2017. LWW. doi: 10.1097/MOG.0000000000000369 28426462 PMC5568812

[7] Teodora Surdea-Blaga, Sebestyen Gheorghe, Czako Zoltan, Hangan Anca, Dumitrascu Dan Lucian, Ismaiel Abdulrahman, et al., Automated chicago classification for esophageal motility disorder diagnosis using machine learning, *Sensors*, vol. 22, no. 14, pp. 5227, 2022. MDPI. doi: 10.3390/s22145227 35890906 PMC9323128

[8] Kou Wenjun, Galal Galal Osama, Klug Matthew William, Mukhin Vladislav, Carlson Dustin A, Etemadi Mozziyar, et al., Deep learning–based artificial intelligence model for identifying swallow types in esophageal high-resolution manometry, *Neurogastroenterology & Motility*, vol. 34, no. 7, pp. e14290, 2022. Wiley Online Library. doi: 10.1111/nmo.14290 34709712 PMC9046460

[9] Kou Wenjun, Carlson Dustin A, Baumann Alexandra J, Donnan Erica N, Schauer Jacob M, Etemadi Mozziyar, et al., A multi-stage machine learning model for diagnosis of esophageal manometry, *Artificial intelligence in medicine*, vol. 124, pp. 102233, 2022. Elsevier. doi: 10.1016/j.artmed.2021.102233 35115131 PMC8817064

[10] Abe Hirofumi, Tanaka Shinwa, Kawara Fumiaki, Toyonaga Takashi, Sakaguchi Hiroya, Nakai Tatsuya, et al., Esophageal motility disorders missed during endoscopy, *Esophagus*, vol. 19, no. 3, pp. 486–492, 2022. Springer. doi: 10.1007/s10388-021-00903-4 35038065

[11] Posner Shai, Mehta Kurren, Parish Alice, Niedzwiecki Donna, Gupta Rajan T, Fisher Deborah A, et al., Esophageal function tests are not associated with barium swallow findings in advanced lung disease, *Dysphagia*, vol. 35, pp. 864–870, 2020. Springer. 32277290 10.1007/s00455-020-10113-2

[12] Shahsavari Dariush, Smith Michael S, Malik Zubair, Parkman Henry P, Hiatal hernias associated with acid reflux: size larger than 2 cm matters, *Diseases of the Esophagus*, vol. 35, no. 8, pp. doac001, 2022. Oxford University Press. doi: 10.1093/dote/doac001 35066592

[13] Howk Amy A, Clifton Matthew S, Garza Jose M, Durham Megan M, Impedance planimetry (EndoFLIP) assisted laparoscopic esophagomyotomy in pediatric population, *Journal of Pediatric Surgery*, vol. 57, no. 12, pp. 1000–1004, 2022. Elsevier. doi: 10.1016/j.jpedsurg.2022.05.004 35659759

[14] Frigo Alessandro, Costantini Mario, Fontanella Chiara Giulia, Salvador Renato, Merigliano Stefano, Carniel Emanuele Luigi, A procedure for the automatic analysis of high-resolution manometry data to support the clinical diagnosis of esophageal motility disorders, *IEEE Transactions on Biomedical Engineering*, vol. 65, no. 7, pp. 1476–1485, 2017. IEEE. doi: 10.1109/TBME.2017.2758441 28976308

[15] Jell Alissa, Kuttler Christina, Ostler Daniel, Norbert Hüser, How to cope with big data in functional analysis of the esophagus, *Visceral Medicine*, vol. 36, no. 6, pp. 439–442, 2020. S. Karger AG. doi: 10.1159/000511931 33447599 PMC7768100

[16] Kou Wenjun, Carlson Dustin A, Baumann Alexandra J, Donnan Erica, Luo Yuan, Pandolfino John E, et al., A deep-learning-based unsupervised model on esophageal manometry using variational autoencoder, *Artificial intelligence in medicine*, vol. 112, pp. 102006, 2021. Elsevier. doi: 10.1016/j.artmed.2020.102006 33581826 PMC7901248

[17] Stefan Lucian Popa, Teodora Surdea-Blaga, Dan Lucian Dumitrascu, Giuseppe Chiarioni, Edoardo Savarino, Liliana David, et al., Automatic Diagnosis of High-Resolution Esophageal Manometry using Artificial Intelligence, *Journal of Gastrointestinal & Liver Diseases*, vol. 31, no. 4, 2022. 10.15403/jgld-4525 36535043

[18] Popa Stefan Lucian, Teodora Surdea-Blaga, Dumitrascu Dan Lucian, Pop Andrei Vasile, Ismaiel Abdulrahman, David Liliana, et al., Gemini-Assisted Deep Learning Classification Model for Automated Diagnosis of High-Resolution Esophageal Manometry Images, *Medicina*, vol. 60, no. 9, pp. 1493, 2024. MDPI. doi: 10.3390/medicina60091493 39336534 PMC11434326

[19] Alexander Geiger, Lars Wagner, Daniel Rueckert, Dirk Wilhelm, Alissa Jell, *Detecting and clustering swallow events in esophageal long-term high-resolution manometry*, 2024.

[20] Qin RuoXi, Wang Zhenzhen, Jiang LingYun, Qiao Kai, Hai Jinjin, Chen Jian, et al., Fine-Grained Lung Cancer Classification from PET and CT Images Based on Multidimensional Attention Mechanism, *Complexity*, vol. 2020, no. 1, pp. 6153657, 2020. Wiley Online Library.

[21] Chen Gongping, Li Lei, Dai Yu, Zhang Jianxun, and Yap Moi Hoon, AAU-net: an adaptive attention U-net for breast lesions segmentation in ultrasound images, *IEEE Transactions on Medical Imaging*, vol. 42, no. 5, pp. 1289–1300, 2022. IEEE. doi: 10.1109/TMI.2022.3226268 36455083

[22] Cao Luyang, Li Jianwei, and Chen Shu, Multi-target segmentation of pancreas and pancreatic tumor based on fusion of attention mechanism, *Biomedical Signal Processing and Control*, vol. 79, p. 104170, 2023. Elsevier. doi: 10.1016/j.bspc.2022.104170

[23] Xu Guohao, Wang Chuantao, Li Zhuoyuan, Zhai Jiliang, and Wang Saishuo, Efficient spine segmentation network based on multi-scale feature extraction and multi-dimensional spatial attention, *International Journal of Imaging Systems and Technology*, vol. 34, no. 2, p. e23046, 2024. Wiley Online Library. doi: 10.1002/ima.23046

[24] Zhang Shidong, He Cong, Wan Zhenzhen, Shi Ning, Wang Bing, Liu Xiuling, et al., Diagnosis of pulmonary tuberculosis with 3D neural network based on multi-scale attention mechanism, *Medical Biological Engineering Computing*, vol. 62, no. 5, 2024. doi: 10.1007/s11517-024-03022-1 38319503

[25] Dequn Zhao, Chunsheng Li, Hongwei Ma, and Suming Zhang, *Retinal image segmentation algorithm based on hybrid pooling and multi-dimensional attention*, in *International Conference on Image*, *Signal Processing*, *and Pattern Recognition (ISPP 2024)*, vol. 13180, pp. 116–121, 2024. SPIE.

[26] Yang Yongquan, Lv Haijun, and Chen Ning, A survey on ensemble learning under the era of deep learning, *Artificial Intelligence Review*, vol. 56, no. 6, pp. 5545–5589, 2023. Springer. doi: 10.1007/s10462-022-10283-5

[27] Zheng Yuchao, Li Chen, Zhou Xiaomin, Chen Haoyuan, Xu Hao, Li Yixin, et al., Application of transfer learning and ensemble learning in image-level classification for breast histopathology, *Intelligent Medicine*, vol. 3, no. 02, pp. 115–128, 2023. Chinese Medical Association Publishing House Co., Ltd. doi: 10.1016/j.imed.2022.05.004

[28] Bayani Azadeh, Hosseini Azamossadat, Asadi Farkhondeh, Hatami Behzad, Kavousi Kaveh, Aria Mehrdad, et al., Identifying predictors of varices grading in patients with cirrhosis using ensemble learning, *Clinical Chemistry and Laboratory Medicine (CCLM)*, vol. 60, no. 12, pp. 1938–1945, 2022. De Gruyter. doi: 10.1515/cclm-2022-0508 35852068

[29] Su Qiaosen, Wang Fengsheng, Chen Dong, Chen Gang, Li Chao, and Wei Leyi, Deep convolutional neural networks with ensemble learning and transfer learning for automated detection of gastrointestinal diseases, *Computers in Biology and Medicine*, vol. 150, p. 106054, 2022. Elsevier. doi: 10.1016/j.compbiomed.2022.106054 36244302

[30] Sivari Esra, Bostanci Erkan, Guzel Mehmet Serdar, Acici Koray, Asuroglu Tunc, and Ayyildiz Tulin Ercelebi, A new approach for gastrointestinal tract findings detection and classification: Deep learning-based hybrid stacking ensemble models, *Diagnostics*, vol. 13, no. 4, p. 720, 2023. MDPI. doi: 10.3390/diagnostics13040720 36832205 PMC9954881

[31] Gunasekaran Hemalatha, Ramalakshmi Krishnamoorthi, Swaminathan Deepa Kanmani, and Mazzara Manuel, GIT-Net: an ensemble deep learning-based GI tract classification of endoscopic images, *Bioengineering*, vol. 10, no. 7, p. 809, 2023. MDPI. doi: 10.3390/bioengineering10070809 37508836 PMC10376874

[32] Ahamed Md Faysal, Md Nahiduzzaman, Islam Md Rabiul, Naznine Mansura, Ayari Mohamed Arselene, Khandakar Amith, et al., Detection of various gastrointestinal tract diseases through a deep learning method with ensemble ELM and explainable AI, *Expert Systems with Applications*, vol. 256, p. 124908, 2024. Elsevier. doi: 10.1016/j.eswa.2024.124908

[33] Kaiming He, Xiangyu Zhang, Shaoqing Ren, and Jian Sun, *Deep residual learning for image recognition*, in *Proceedings of the IEEE conference on computer vision and pattern recognition*, pp. 770–778, 2016.

[34] Andrew Howard, Mark Sandler, Grace Chu, Liang-Chieh Chen, Bo Chen, Mingxing Tan, et al., *Searching for mobilenetv3*, in *Proceedings of the IEEE/CVF international conference on computer vision*, pp. 1314–1324, 2019.

[35] Christian Szegedy, Wei Liu, Yangqing Jia, Pierre Sermanet, Scott Reed, Dragomir Anguelov, et al., *Going deeper with convolutions*, in *Proceedings of the IEEE conference on computer vision and pattern recognition*, pp. 1–9, 2015.

[36] Mingxing Tan, *Efficientnet: Rethinking model scaling for convolutional neural networks*, *arXiv preprint arXiv:1905.11946*, 2019.

[37] Gao Huang, Zhuang Liu, Laurens Van Der Maaten, and Kilian Q. Weinberger, *Densely connected convolutional networks*, in *Proceedings of the IEEE conference on computer vision and pattern recognition*, pp. 4700–4708, 2017.

[38] Karen Simonyan and Andrew Zisserman, *Very deep convolutional networks for large-scale image recognition*, *arXiv preprint arXiv:1409.1556*, 2014.

[39] Ze Liu, Yutong Lin, Yue Cao, Han Hu, Yixuan Wei, Zheng Zhang, et al., *Swin transformer: Hierarchical vision transformer using shifted windows*, in *Proceedings of the IEEE/CVF international conference on computer vision*, pp. 10012–10022, 2021.

[40] Alexey Dosovitskiy, *An image is worth 16x16 words: Transformers for image recognition at scale*, *arXiv preprint arXiv:2010.11929*, 2020.

[41] Wang Wenhai, Xie Enze, Li Xiang, Fan Deng-Ping, Song Kaitao, Liang Ding, et al., Pvt v2: Improved baselines with pyramid vision transformer, *Computational Visual Media*, vol. 8, no. 3, pp. 415–424, 2022. Springer. doi: 10.1007/s41095-022-0274-8

[42] Ao Wang, Hui Chen, Zijia Lin, Jungong Han, and Guiguang Ding, *RepViT: Revisiting Mobile CNN From ViT Perspective*, *IEEE*, 2023.

